# On clustering for cell-phenotyping in multiplex immunohistochemistry (mIHC) and multiplexed ion beam imaging (MIBI) data

**DOI:** 10.1186/s13104-022-06097-x

**Published:** 2022-06-20

**Authors:** Souvik Seal, Julia Wrobel, Amber M. Johnson, Raphael A. Nemenoff, Erin L. Schenk, Benjamin G. Bitler, Kimberly R. Jordan, Debashis Ghosh

**Affiliations:** 1 Department of Biostatistics and Informatics, University of Colorado CU Anschutz Medical Campus, Aurora, Colorado USA; 2 Department of Medicine, School of Medicine, University of Colorado CU Anschutz Medical Campus, Aurora, Colorado USA; 3 Division of Medical Oncology, School of Medicine, University of Colorado CU Anschutz Medical Campus, Aurora, Colorado USA; 4 Department of Obstetrics and Gynecology, School of Medicine, University of Colorado CU Anschutz Medical Campus, Aurora, Colorado USA; 5 Department of Immunology and Microbiology, School of Medicine, University of Colorado CU Anschutz Medical Campus, Aurora, Colorado USA

**Keywords:** Multiplex tissue imaging, Cell phenotyping, Vectra polaris, MIBI, Semi-supervised Learning

## Abstract

**Objective:**

Multiplex immunohistochemistry (mIHC) and multiplexed ion beam imaging (MIBI) images are usually phenotyped using a manual thresholding process. The thresholding is prone to biases, especially when examining multiple images with high cellularity.

**Results:**

Unsupervised cell-phenotyping methods including PhenoGraph, flowMeans, and SamSPECTRAL, primarily used in flow cytometry data, often perform poorly or need elaborate tuning to perform well in the context of mIHC and MIBI data. We show that, instead, semi-supervised cell clustering using Random Forests, linear and quadratic discriminant analysis are superior. We test the performance of the methods on two mIHC datasets from the University of Colorado School of Medicine and a publicly available MIBI dataset. Each dataset contains a bunch of highly complex images.

**Supplementary Information:**

The online version contains supplementary material available at 10.1186/s13104-022-06097-x.

## Introduction

Several multiplex tissue imaging technologies have recently been developed for probing single-cell spatial biology, including multiparameter immunofluorescence [1], multiplex immunohistochemistry (mIHC) [2] and multiplexed ion beam imaging (MIBI) [3].

The spatial capabilities of these new technologies offer up the potential for researchers to develop a novel understanding of the biological mechanisms underlying cellular and protein interactions in a wide array of scientific contexts. These platforms are rapidly developing and all produce data of a similar structure: two dimensional images of tissue at the resolution of cells and nuclei, where proteins in the sample have been labeled with antibodies called “markers” that attach to cell membranes.

mIHC data collected from platforms such as Vectra 3 or Vectra Polaris typically have 6–8 markers [4], while some platforms like MILAN can have around 40 markers [5]. MIBI images have 40–50 markers [3].

mIHC and MIBI technologies have many data pre-processing and analyses steps that have not yet been uniformly implemented. Cell-phenotyping, defined as identification of cell populations based on marker expression, is a challenging process in this context. In most of the current cell-phenotyping approaches, researchers require to manually set a threshold intensity value for every marker, and

cells are then phenotyped based on the binarized expression of all the markers. For example, CD4 T cells are positive for markers CD3 and CD4 and negative for CD8. This manual phenotyping (gating) approach is cumbersome for high parameter panels and depends on the reliability and expert knowledge of the user selecting positive cells or choosing thresholds, which may differ between users. Thus, manual gating is not only prone to human error but also time consuming and costly. Algorithms have already been developed to tackle these same phenotyping issues for multiplex technologies that analyze single cells in a liquid suspension without spatial resolution, namely flow and mass cytometry [6]. In particular, automated gating methods using machine learning algorithms have become more and more popular as the number of analyzed parameters has increased [7].

Our aim in this paper is to compare automated cell-phenotyping algorithms in the context of mIHC and MIBI datasets. We adapt approaches originally developed for two non-spatial technologies, flow and mass cytometry, and test our algorithms on two mIHC datasets [4, 8] obtained from the University of Colorado School of Medicine and one publicly available MIBI dataset [9].

## Main text

### **Existing phenotyping algorithms**

#### Unsupervised learning algorithms

Unsupervised cell-phenotyping algorithms partition cells into different classes based on their multiplex marker expression without using any prior knowledge [10]. These methods are initially unbiased and usually time and memory efficient as well. In addition, novel cell types and populations can be discovered by not biasing clustering algorithms with prior information about marker expression. However, these methods suffer from several major limitations. For example, once the cells have been classified by an unsupervised algorithm, researchers manually gate the obtained classes to compare meaningful cell types (e.g. CD4 T cell, CD68+ macrophages etc.). This step can be cumbersome and again prone to human error. PhenoGraph [11], flowMeans [12] and SamSPECTRAL [13] are some of the most popular unsupervised cell-phenotyping algorithms [6, 7].

#### Semi-supervised learning algorithms

Semi-supervised cell-phenotyping approaches typically involve building a predictive model using multiplex marker expression from a subset of cells in a dataset, called the training set, that have been manually phenotyped [14]. The built models are then used to phenotype the remaining cells, or the test set. Unlike unsupervised methods, the cells in this case are directly assigned to existing phenotypes which obviates the problem of matching arbitrary clusters to meaningful cell types. One can argue that the first step of manually phenotyping cells in the training set is subjected to human error. However, the size of the training set is usually just a fraction of the full dataset. Therefore, ensuring the purity of manual phenotyping of the training dataset should be easy relative to manually phenotyping all of the data; though this remains a practical limitation for all current approaches.

DeepCyTOF [15], CyTOF linear classifier [16] and ACDC [17] are popular semi-supervised methods in flow and mass cytometry [7]. CyTOF linear classifier, which is based on linear discriminant analysis (LDA), has been shown to outperform more complex algorithms like DeepCyTOF, ACDC on several CyTOF datasets [7, 16]. All the above methods are briefly described further in Additional file 1: Table S1.

LDA assumes that the data has equal variance across groups and is normally distributed. Though these assumptions may hold for CyTOF data, in mIHC datasets both assumptions are violated. To address these problems, we consider more general machine-learning algorithms such as quadratic discriminant analysis (QDA) [18] and Random Forest [19]. QDA is similar to LDA but does not require equal variance across groups. The decision tree-based Random Forest method is robust for non-normal data and has several additional advantages demonstrated by [20]; these include minimal tuning parameters, excellent off-the-shelf prediction, honest estimates of classification through out-of-bag samples, and stable prediction behavior. Therefore, in the context of mIHC and MIBI data, we propose to use Random Forest and compare its performance with LDA and QDA.

### **Datasets**

Our analysis incorporated three multiplex tissue imaging datasets: an ovarian cancer dataset [8] acquired on the mIHC Vectra Polaris platform (Akoya Biosciences), a lung cancer dataset [4] acquired on the mIHC Vectra 3.0 system (Akoya Biosciences), and a breast cancer dataset [9] collected on the MIBI platform (IonPath, Inc). The two mIHC datasets were segmented and phenotyped using inForm (v2.4.8, Akoya Biosciences), commercially available software for Vectra data [21], and the MIBI dataset was phenotyped in MATLAB using deep learning-based methods [9]. For each cell, the expression data is available for multiple markers. The datasets are described in detail below and Table 1 lists the overall distribution of the cell types in different datasets.

#### mIHC ovarian cancer dataset

There are 302,147 cells from 132 subjects. There are five different cell types: CD19+, CD3+/CD8-, CD3+/CD8+, CD68+, CK+/Ki67+. There are six markers, CD19, CD3, CK, CD8, Ki67, CD68 observed in each of the cells. More details on this data can be found at [8].

#### mIHC lung cancer dataset

There are 1,590,327 cells from 153 subjects each with 3-5 images (in total, 761 images). There are six different cell types: CD14+, CD19+, CD4+, CD8+, CK+, Other+ (meaning they do not belong to any of the indicated phenotypes). There are five markers, CD19, CD3, CK, CD8, CD14. More details on this data can be found at [4].

#### MIBI breast cancer dataset

The triple-negative breast cancer (TNBC) MIBI dataset [9] has 201,656 cells from 43 subjects and one image per subject. It has six different cell groups: Immune, Endothellial, Mesenchymal-like, Tumor, Keratin-positive tumor and Unidentified. There are 44 markers available, such as CD3, CD8, CD63, Ki67, and Vimentin.

**Table 1:** The frequency of cells belonging to different cell types in different datasets

Dataset	Cell type	Total cells
mIHC ovarian cancer	CD19+	15267 (5%)
	CD3+/CD8-	15952 (5.3%)
	CD3+/CD8+	41008 (13.6%)
	CD68+	57632 (19.1%)
	CK+/Ki67+	172288 (57%)
mIHC lung cancer	CD14+	175878 (11.1 %)
	CD19+	154045 (9.7 %)
	CD4+	232878 (14.6 %)
	CD8+	124102 (7.8 %)
	CK+	594140 (37.4 %)
	Other+	309284 (19.4 %)
MIBI breast cancer	Unidentified	1839 (1 %)
	Immune	83336 (41.3 %)
	Endothelial	2089 (1 %)
	Mesenchymal-like	8479 (4.2 %)
	Tumor	3177 (1.6 %)
	Keratin-positive tumor	102736 (50.9 %)

### **Results**

We primarily focused on the semi-supervised methods in this paper. First, we briefly highlighted some of the major problems of the unsupervised methods using the mIHC lung cancer dataset. Then, we compared the usability and performance of Random Forest with LDA and QDA in all three datasets.

#### Unsupervised methods

**Fig. 1:**
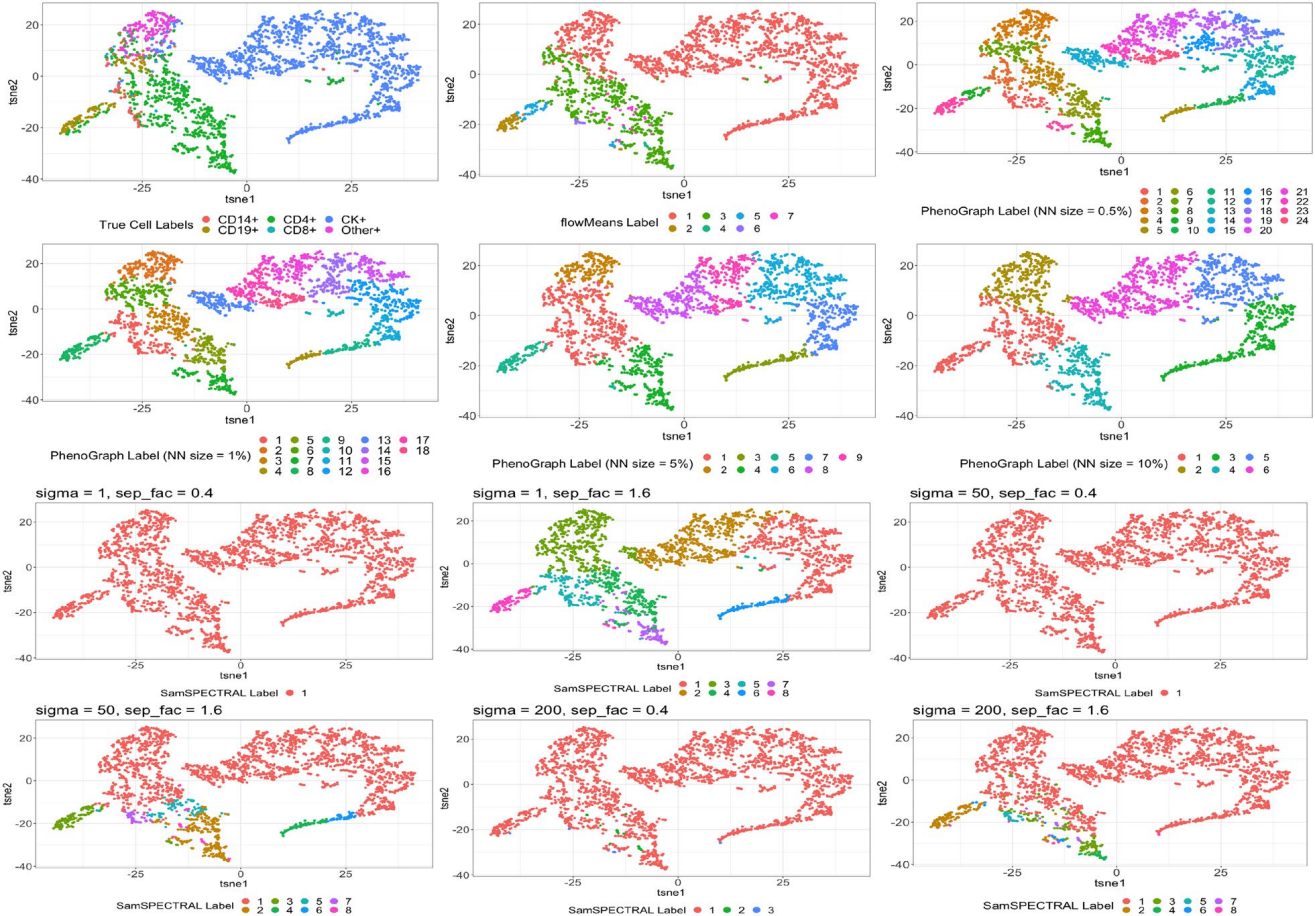
Comparison of the cell labels estimated by PhenoGraph, flowMeans and SamSPECTRAL with the true cell labels for a particular subject. The top two rows show the scatter-plot of TSNE1 and TSNE2 for different cells colored by three different labels, true labels, estimated labels using flowMeans and estimated labels using PhenoGraph for varying number of nearest neighbor(NN)-sizes. The bottom two rows show the scatter-plot of TSNE1 and TSNE2 for different cells colored by the estimated labels using SamSPECTRAL for different values (from low to high) of sigma and separation factor (sep_fac)

In the mIHC lung cancer dataset, we clustered the cells of one subject at a time using the unsupervised methods, PhenoGraph, SamSPECTRAL and flowMeans. T-distributed stochastic neighbor embedding (t-SNE) [22] has been used by researchers to visualize high-dimensional data in various contexts including flow and mass cytometry [23, 24]. In Fig. 1, for a particular subject, we compared the true cell labels with the labels estimated using the unsupervised methods, overlaid on the first two t-SNEs of the marker data. PhenoGraph and SamSPECTRAL depend on the choice of several pre-specified hyper-parameters. PhenoGraph depends on the number of nearest neighbors (NN’s), whereas SamSPECTRAL depends on two quantities known as sigma and separation factor. For PhenoGraph, we considered 4 different NN sizes, namely $0.5 \%, 1\%, 5\%$ and $10 \%$ of the total number of cells. For most of the subjects, including the one depicted in Fig. 1, PhenoGraph classified the cells into a large number of clusters when NN size was small. For larger NN sizes, PhenoGraph generated around 6 clusters but it would require additional evaluation of the clusters to properly map them with true and meaningful cell-labels. Similarly, the performance of SamSPECTRAL was highly variable depending on the input values of the tuning parameters, and none of the combinations yielded clusters that remotely resembled the true cell labels. On the other hand, the result from flowMeans looked fairly close to the true cell-labels and it would require the least amount of post-clustering evaluation compared to the previous two methods.

We should reiterate that we did not provide a systematic comparison of the unsupervised methods here. Our goal was to briefly highlight the major difficulties with the unsupervised methods, namely that the results may vary significantly based on the choice of the tuning parameters and also, require additional evaluation of the obtained clusters for a meaningful mapping with the true cell-phenotypes.

#### Semi-supervised methods

For each dataset, we randomly selected *m* training images (out of the total size, *M*) to train the models on and evaluated their performance on the remainder of the images. We varied *m* and for every choice of *m*, we considered 5 repetitions. Results were aggregated across repetitions and summarized by prediction accuracy, adjusted rand index (ARI), and normalized mutual information (NMI).

#### mIHC ovarian cancer dataset

We considered four training set-sizes (*m*) which were fractions of the total size *M*, $m = 7$ ($5\%$), 13 ($10\%$), 20 ($15\%$), and 26 ($20\%$). Table 2 lists the mean (and standard deviation) of prediction accuracy, ARI, and NMI. Even for the smallest *m*, all three methods performed well, with Random Forest having the highest mean prediction accuracy, ARI, and NMI. Random Forest also had significantly lower standard deviation which accentuated its high robustness. As *m* increased, prediction accuracy, ARI, and NMI marginally improved for all three methods.

#### mIHC lung cancer dataset

We considered *m* to be, 4 ($0.5\%$), 8 ($1\%$), 15 ($2\%$), 23 ($3\%$) and 76 ($10\%$). Random Forest again outperformed LDA and QDA (Table 2). However, the prediction accuracy was significantly lower for the smaller training set-sizes. Random Forest’s performance steadily improved as the training set-size (*m*) increased, whereas for LDA and QDA, the performance stayed nearly the same. We noticed a dip in the overall performance of all the methods in this dataset compared to the ovarian cancer dataset. Further details are provided in the Additional file 1. Additional file 1: Figs. S1–3 respectively show the accuracy of Random Forest for predicting every cell type, the proportion of predicted cell types vs every known cell type, and the overall intensity of CD19 marker in different images.

**Table 2:** Prediction accuracy, ARI and NMI mean (± standard deviation) for different training set sizes in mIHC ovarian and lung cancer datasets and MIBI breast cancer dataset

Dataset	Training size	Method	Accuracy	ARI	NMI
mIHC ovarian cancer	5$\%$	Random Forest	0.944 ± 0.004	0.888 ± 0.007	0.783 ± 0.010
		LDA	0.899 ± 0.017	0.779 ± 0.047	0.642 ± 0.051
		QDA	0.909 ± 0.007	0.821 ± 0.023	0.699 ± 0.018
	10$\%$	Random Forest	0.949 ± 0.002	0.896 ± 0.004	0.795 ± 0.006
		LDA	0.889 ± 0.010	0.748 ± 0.027	0.609 ± 0.028
		QDA	0.919 ± 0.003	0.842 ± 0.007	0.720 ± 0.008
	15$\%$	Random Forest	0.951 ± 0.002	0.899 ± 0.003	0.802 ± 0.006
		LDA	0.898 ± 0.006	0.772 ± 0.018	0.633 ± 0.020
		QDA	0.920 ± 0.001	0.848 ± 0.005	0.724 ± 0.006
	20$\%$	Random Forest	0.952 ± 0.002	0.902 ± 0.002	0.806 ± 0.006
		LDA	0.899 ± 0.007	0.774 ± 0.018	0.634 ± 0.023
		QDA	0.922 ± 0.001	0.853 ± 0.003	0.727 ± 0.006
mIHC lung cancer	0.5$\%$	Random Forest	0.734 ± 0.179	0.575 ± 0.022	0.426 ± 0.018
		LDA	0.668 ± 0.052	0.413 ± 0.102	0.363 ± 0.070
		QDA	0.669 ± 0.048	0.459 ± 0.076	0.365 ± 0.036
	1$\%$	Random Forest	0.755 ± 0.057	0.594 ± 0.021	0.450 ± 0.013
		LDA	0.704 ± 0.057	0.486 ± 0.116	0.395 ± 0.068
		QDA	0.692 ± 0.040	0.482 ± 0.067	0.387 ± 0.031
	2$\%$	Random Forest	0.768 ± 0.009	0.608 ± 0.016	0.468 ± 0.011
		LDA	0.686 ± 0.063	0.440 ± 0.133	0.374 ± 0.083
		QDA	0.696 ± 0.019	0.472 ± 0.030	0.387 ± 0.010
	3$\%$	Random Forest	0.777 ± 0.002	0.620 ± 0.008	0.480 ± 0.005
		LDA	0.674 ± 0.064	0.424 ± 0.134	0.355 ± 0.084
		QDA	0.687 ± 0.024	0.452 ± 0.044	0.373 ± 0.024
	10$\%$	Random Forest	0.805 ± 0.001	0.665 ± 0.003	0.524 ± 0.003
		LDA	0.709 ± 0.008	0.500 ± 0.024	0.393 ± 0.011
		QDA	0.705 ± 0.011	0.475 ± 0.027	0.386 ± 0.011
MIBI breast cancer	5$\%$	Random Forest	0.951 ± 0.016	0.869 ± 0.037	0.772 ± 0.055
		LDA	0.781 ± 0.135	0.618 ± 0.111	0.47 ± 0.065
	10$\%$	Random Forest	0.971 ± 0.010	0.915 ± 0.027	0.853 ± 0.04
		LDA	0.836 ± 0.038	0.632 ± 0.045	0.492 ± 0.045
	20$\%$	Random Forest	0.983 ± 0.002	0.948 ± 0.008	0.903 ± 0.011
		LDA	0.877 ± 0.010	0.714 ± 0.020	0.569 ± 0.018

#### MIBI breast cancer dataset

We considered three values of *m*, 2 ($5\%$), 4 ($10\%$) and 8 ($20\%$). Even with the smallest *m*, Random Forest achieved great prediction accuracy (Table 2). LDA was consistently poorer than Random Forest but its accuracy increased steadily as *m* increased. We did not report the performance of QDA for this dataset since it often encountered an error due to “rank deficiency”, especially for small training sizes (refer to the Additional file 1: Table S2).

## Limitations

We have noticed that cells of certain types can get incorrectly phenotyped if the corresponding markers are not informative enough. For example, in some subjects from the lung cancer dataset, CD19 marker intensity is not distinctive across different cell types which makes identifying CD19+ cells hard. It shall also be kept in mind that the mIHC datasets we analyzed were originally phenotyped using the inForm software. It is a possibility that the original phenotyping was inaccurate and thus our “ground truth”itself was biased.

The run-time comparison of the methods are provided in Additional file 1: Table S2. We noted that LDA and QDA both took fractions of the time taken by Random Forest model. In the MIBI dataset, QDA encountered convergence error for some particular choices of the training set, especially with a smaller training set-size. Therefore, when there are large numbers of markers and cells, we recommend using LDA over Random Forest which would potentially sacrifice some degree of accuracy but be much more scalable. Besides, it should also be kept in mind that the semi-supervised methods in general can be unreliable for detecting rare cell-populations which would ideally require a specialist’s manual evaluation of the marker expression-profiles. In this study, all the datasets we considered, had 5–6 cell types. In future, we will check the applicability of the methods on multiplex imaging datasets which have a larger number of cell types.

## Supplementary Information

**Additional file 1. **Here, we provide a section explaining the overall dip in the performance of the methods in the mIHC lung cancer dataset.** Figure S1–3.** focus on the mIHC lung cancer dataset, and respectively show the scatter-plot of accuracy of Random Forest for predicting every cell type, the bar-plot of pro-portion of predicted cell types vs every known cell type, and the ridge-plot of overall CD19 marker intensity in the cells of different images.** Table S1, 2.** respectively list the summary of a few existing methods and the run-times of the methods in different datasets.

## Data Availability

The MIBI breast cancer dataset used in the paper can be found at this link, https://www.angelolab.com/mibi-data. The mIHC datasets are available from the corresponding author on reasonable request. Our methods can be found as a *R* package named as VectraMIBI at this link. The package builds a Random Forest model on a given training dataset, and uses predictions from that model to annotate (phenotype) the cells of a test dataset. The package also provides visualization tools including heat-maps of the mean marker intensity over different cell types and image specific ridge-plots of the marker intensity for different cell types for basic exploration of the training dataset.

## Abbreviations

mIHC: Multiplex Immuno Histochemistry
MIBI: Multiplex Ion Beam Imaging
LDA: Linear Discriminant Analysis
QDA: Quadratic Discriminant Analysis
