# Supplementary material for: On clustering for cell-phenotyping in multiplex immunohistochemistry (mIHC) and multiplexed ion beam imaging (MIBI) data
Source: BMC Res Notes. 2022 Jun 20;15:215. doi: 10.1186/s13104-022-06097-x (PMC9208090; doi:10.1186/s13104-022-06097-x)
Supplement: Supplementary file 1 — Additional file 1. Here, we provide a section explaining the overall dip in the performance of the methods in the mIHC lung cancer dataset. Figure S1–3. focus on the mIHC lung cancer dataset, and respectively show the scatter-plot of accuracy of Random Forest for predicting every cell type, the bar-plot of pro-portion of predicted cell types vs every known cell type, and the ridge-plot of overall CD19 marker intensity in the cells of different images. Table S1, 2. respectively list the summary of a few existing methods and the run-times of the methods in different datasets. [file 13104_2022_6097_MOESM1_ESM.pdf]

Supplementary material

Souvik Seal, Julia Wrobel, Amber M. Johnson, Raphael A. Nemenoff, Erin L. Schenk, Benjamin G. Bitler, Kimberly R. Jordan and Debashis Ghosh

June 3, 2022

1 Investigating the poor performance of the methods in the mIHC lung cancer dataset

To investigate the dip in overall performance in the mIHC lung cancer dataset compared to the ovarian cancer dataset, we looked at the cell type specific prediction accuracy for a fixed training set. In Figure S1, we show the scatter-plot of the prediction accuracy for the six different cell types in all the 761 images. For cell type CD19+, we observed that the prediction accuracy was quite low (median accuracy 0.298), especially in the images where the CD19+ cell frequency was low. Figure S2 shows how many of the cells of a particular type were actually predicted to be of that type. For example, from Figure S2 (b), we noticed that a significant number of CD19+ cells got assigned to types: CD4+, Other+. Since we would expect the CD19 marker staining intensity to be the instrumental variable in separating CD19+ cells from the other cell types, we inspected its distribution in the images for which CD19+ prediction accuracy was above 0.9 and the images for which CD19+ prediction accuracy was less than 0.25, illustrated in two ridge plots in Figure S3. Figure S3 (a) shows the distribution of the CD19 marker staining intensity (color coded by original cell type) of the cells from four images for which CD19+ prediction accuracy is higher than 0.9 and Figure S3 (b) shows the ridge plot of CD19 marker staining intensity (color coded by original cell type) of the cells from five images for which CD19+ prediction accuracy is lower than 0.25. We noticed that in the high-accuracy images, the CD19+ cells have distinctively high staining intensities for the CD19 marker compared to the cells of other types (Figure S3 (a)). However, the CD19 marker intensity is not distinctively different across the cell types in any of the low-accuracy images (Figure S3 (b)). We concluded that the low and non-differential

staining intensity of the CD19 marker across different cell types is the cause of the poor prediction accuracy of CD19+ cells in several of the images.

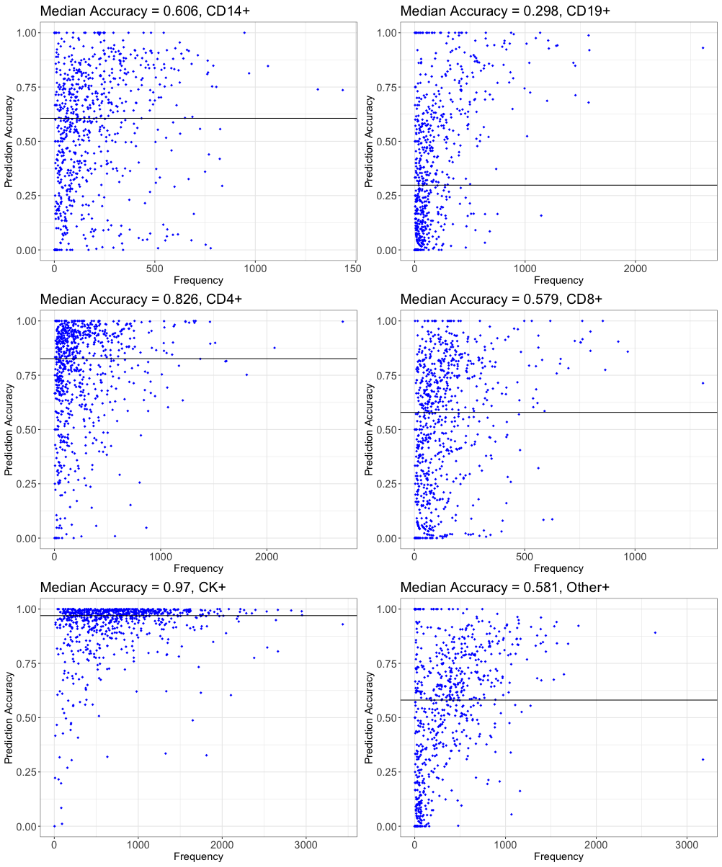

Figure S1: In the mIHC lung cancer dataset, for each cell type, we show the scatter-plot of its frequency (number of cells) in each of the images and the corresponding prediction accuracy. Median accuracy for each of the cell type is mentioned above the corresponding sub-plot. For cell type CD19+, the prediction accuracy is quite low, especially in the images where CD19+ cell frequency is low.

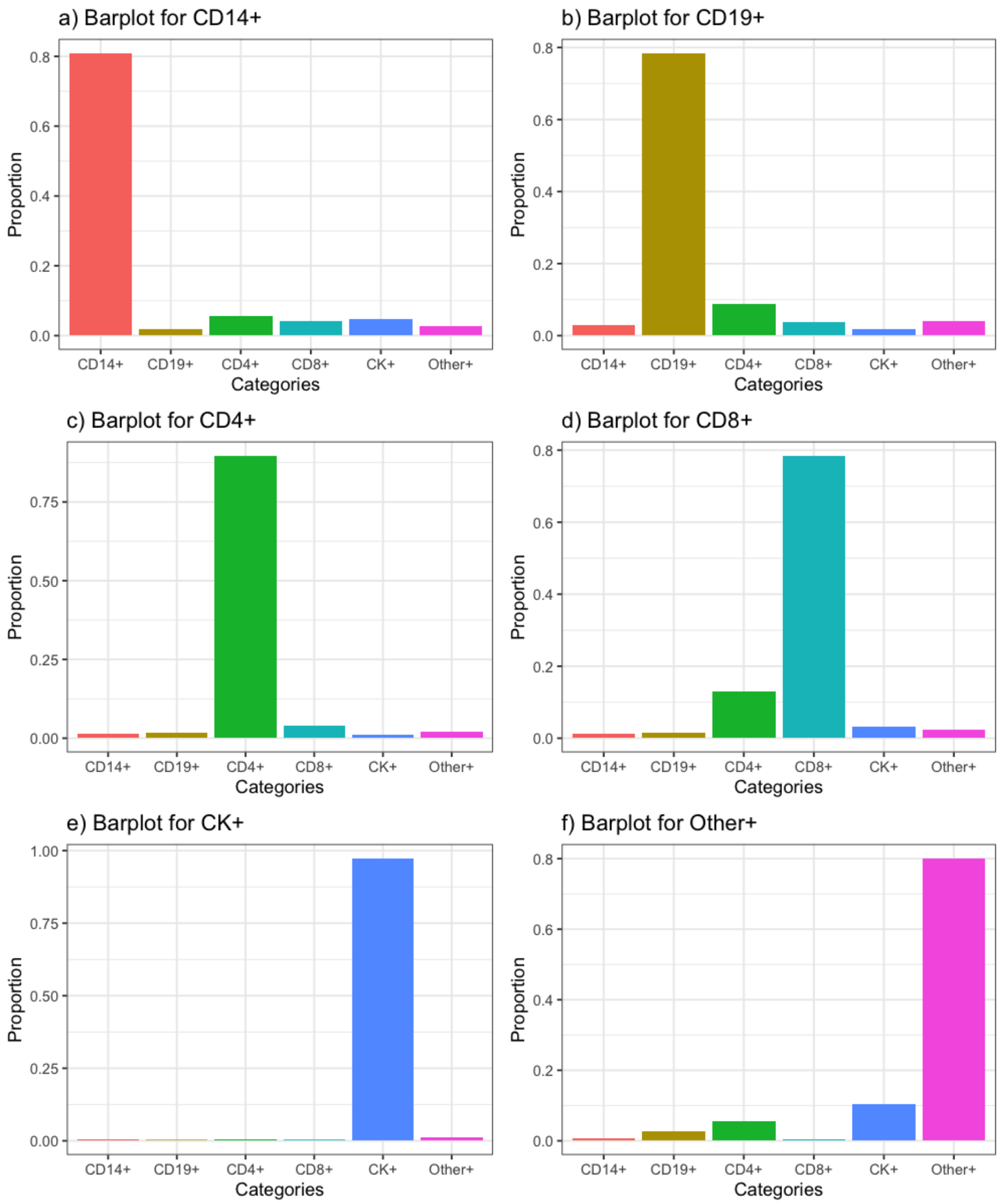

Figure S2: The bar-plots show how many of the cells of a particular type are actually getting predicted to be in that category in the mIHC lung cancer dataset. For example, (b) shows that a significant number of CD19+ cells get assigned to CD4+ and Other+ categories.

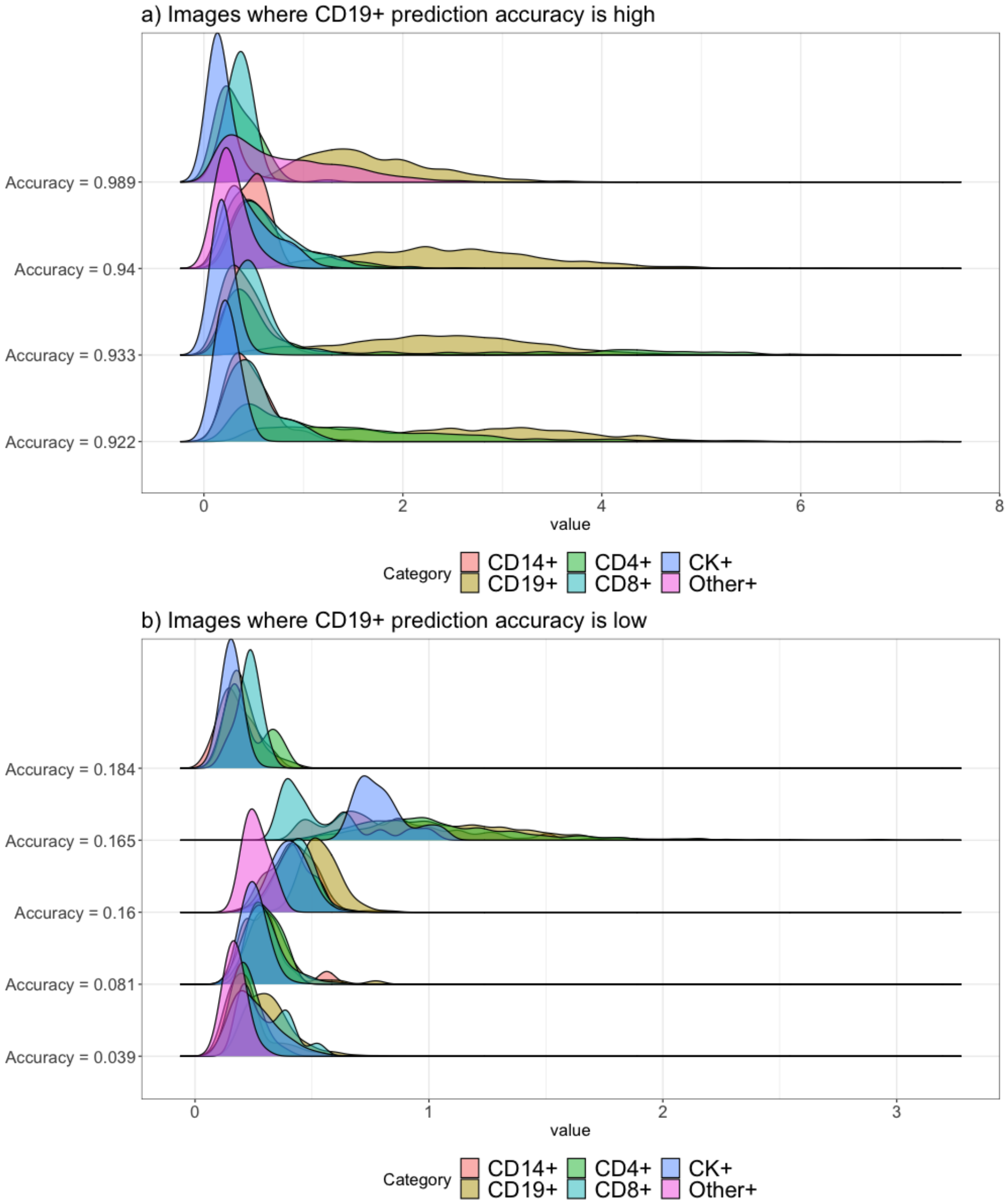

Figure S3: For the mIHC lung cancer dataset, (a) shows the ridge plot of mean CD19 marker intensity of cells (color coded by original phenotype) from some of the images in which CD19+ prediction accuracy is greater than 0.9. (b) shows the ridge plot of mean CD19 marker intensity of cells from some of the images in which CD19+ prediction accuracy is less than 0.25.

Table S1: Brief description of some of the existing methods in the field of Flow and Mass Cytometry.

Type	Methods	Implementation tools	Brief Description
Unsupervised	PhenoGraph	R and Python	Detection of k-nearest neighbors of each cell, Jaccard similarity coefficient as connectivity, community detection based on connection density
	SamSPECTRAL	R	Employ a careful data reduction scheme if the sample size is huge, apply Spectral clustering on the reduced (or, full) data
	flowMeans	R	Modified k-means clustering, merging clusters by distance metrics
Semi-supervised	LDA	R and MATLAB	Linear discriminant analysis with training datasets
	DeepCyTOF	Python	Uses deep learning techniques on training dataset to build up a model that can be used for prediction
	ACDC	Python	Uses a cell type-marker table to determine landmark points based on which classification via random walks is performed

Table S2: Computation time (in minutes) across methods, datasets, and training set sizes. All the methods were run on a *Mac* system with 32 GB DDR4 RAM and 2.4 GHz 8-Core Intel Core i9 processor. For the MIBI dataset, 'X' means that QDA failed to converge for some choices of the training set and thus, was not considered. The error arose if in the training dataset, the sample covariance matrix between the markers for one of the the six cell types became rank deficient.

Dataset	Training size	Random Forest	LDA	QDA
mIHC ovarian cancer	5%	6.15	0.798	0.751
	10%	11.01	1.059	1.029
	15%	19.86	1.453	1.395
	20%	23.49	1.585	1.516
mIHC lung cancer	0.5%	4.04	0.82	0.77
	1%	7.55	0.86	0.83
	2%	13.75	1.16	1.05
	3%	21.04	1.37	1.29
	10%	87.02	2.80	2.66
MIBI breast cancer	5%	13.54	0.001	X
	10%	30.93	0.001	X
	20%	68.87	0.002	X
